# Practical Application of Methanol-Mediated Mutualistic Symbiosis between *Methylobacterium* Species and a Roof Greening Moss, *Racomitrium japonicum*

**DOI:** 10.1371/journal.pone.0033800

**Published:** 2012-03-29

**Authors:** Akio Tani, Yuichiro Takai, Ikko Suzukawa, Motomu Akita, Haruhiko Murase, Kazuhide Kimbara

**Affiliations:** 1 Institute of Plant Science and Resources, Okayama University, Okayama, Japan; 2 Research Institute of Environment, Agriculture and Fisheries, Osaka Prefectural Government, Osaka, Japan; 3 Meiho-Construction Incorporation, Shiga, Japan; 4 Faculty of Biological Engineering, Kinki University, Wakayama, Japan; 5 Graduate School of Agriculture and Biological Sciences, Osaka Prefecture University, Osaka, Japan; 6 Faculty of Engineering, Shizuoka University, Shizuoka, Japan; Belgian Nuclear Research Centre SCK/CEN, Belgium

## Abstract

Bryophytes, or mosses, are considered the most maintenance-free materials for roof greening. *Racomitrium* species are most often used due to their high tolerance to desiccation. Because they grow slowly, a technology for forcing their growth is desired. We succeeded in the efficient production of *R. japonicum* in liquid culture. The structure of the microbial community is crucial to stabilize the culture. A culture-independent technique revealed that the cultures contain methylotrophic bacteria. Using yeast cells that fluoresce in the presence of methanol, methanol emission from the moss was confirmed, suggesting that it is an important carbon and energy source for the bacteria. We isolated *Methylobacterium* species from the liquid culture and studied their characteristics. The isolates were able to strongly promote the growth of some mosses including *R. japonicum* and seed plants, but the plant-microbe combination was important, since growth promotion was not uniform across species. One of the isolates, strain 22A, was cultivated with *R. japonicum* in liquid culture and in a field experiment, resulting in strong growth promotion. Mutualistic symbiosis can thus be utilized for industrial moss production.

## Introduction

People living in large urban areas in mid- and low latitudes endure high summer temperatures. The temperature in urban areas rises much higher than in surrounding areas, making these areas a so-called heat island. This is largely due to the heat stored in pavement and the concrete of buildings, the heavy use of air conditioners, and the heat emitted by vehicles. Covering buildings with plants is an effective solution to this problem. Besides enhancing aesthetics and improving air quality, green roofs decrease the energy consumption of buildings by reducing the temperature of roof surfaces [1].

Roof greening should be done accordingly, on a case by case basis. For most buildings that are small, have a low load capacity or have low accessibility, maintenance-free roof greening is desired. Roof greening technology using mosses is increasingly attracting attention, because it requires the least amount of soil, water, fertilizer, and maintenance. In addition, it is also applicable for wall greening. However, the production capacity does not meet the demand due to the slow growth of mosses.

*Racomitrium* species are usually found on sunny, well-drained sand or rocks [2]. As with many mosses, this species is tolerant to extreme desiccation. This trait enables the application of the moss for maintenance-free roof greening. We have succeeded in factory-level production of *R. japonicum*. The moss is grown in a liquid culture (moss dry weight 3.5 kg in a 1000-L tank) that enhances branching of the plant, which is then shaped into carpets and grown in a greenhouse. This results in 2–3 times faster growth than on sand. Since the liquid culture is not sterilized, however, sometimes the culture becomes unstable, apparently resulting from bacterial invasion. Thus understanding the microbial consortium in the liquid culture is a key to stabilize cultivation of the moss.

We isolated various microorganisms from liquid culture samples, and showed that they affect moss growth in vitro [3]. Because we used a cultivation-dependent technique, however, the analysis did not necessarily reflect the true microbial community structure in the liquid culture. In this paper, we applied a cultivation-independent technique to reveal the microbial community structure in the liquid culture samples, and isolated microorganisms able to promote moss growth, which enhances large-scale moss production as well as crop production.

## Methods

### Sample collection and moss liquid culture condition

The samples of adult gametophytes and liquid cultures of *R. japonicum* used in this study were the same as previously reported [3]. In brief, moss samples collected in Yamagata (fresh weight, 10 g; weight after drying overnight at 80°C, 2 g) were washed 5 times with distilled water to remove soil and inoculated in 4 L of unsterilized liquid culture medium in a 5-L culture bottle. Since the culture medium evaporated during cultivation, medium was added occasionally to fill the flasks to the original volume. Four different culture conditions were used: (A) Hyponex (straight type, N-P-K = 0.1/0.2/0.1, HYPOneX Japan Co., Osaka, Japan) diluted 10,000-fold with tap water; evaporation loss mentioned above was compensated using the same medium, (B) the same as condition A but evaporation loss was compensated with addition of tap water, (C) Hyponex diluted 10,000-fold with distilled water; evaporation loss was compensated using the same medium, (D) the same as condition C but evaporation loss was compensated with addition of distilled water. The pH in all cases was 7.2, and it was maintained with 1 M hydrochloric acid and/or 1 M sodium hydroxide. Air was bubbled into the flask at 3 L/min. The moss was allowed to grow under a continuous photon flux density of 100 µmol/m^2^/s under a fluorescent lamp at 20°C for 8 weeks. At 1, 4, and 7 weeks, 100-mL samples were taken and frozen at −20°C until use.

### Denaturing gradient gel electrophoresis (DGGE)

Samples of the 12 liquid cultures of *R. japonicum* (conditions A to D and 1, 4, 7 weeks, as described above, 10 mL) were centrifuged to collect bacterial cells. The cells were suspended in SNET buffer (20 mM Tris-HCl, pH 8.0, 5 mM EDTA, 400 mM NaCl, 0.3% SDS, 200 µg/mL proteinase K) and used for PCR. PCR was done with NovaTaq DNA polymerase (Shimadzu, Kyoto, Japan) and with the primer pair F338-GC (5′-CGCCCGCCGCGCGCGGCGGGCGGGGCGGGGGCACGGGGGGACTCCTACGGGAGGCAGCAG-3′) and R517 (5′-ATTACCGCGGCTGCTGG-3′) [4]. The amplicons (positions 341 to 534, corresponding to the 16S rRNA gene of *Escherichia coli*) were separated in a 40 to 60% denaturing gradient of 7 M urea and 40% (vol/vol) formamide at 60°C [4] using the DCode system (Bio-Rad Japan, Tokyo, Japan). The DNA bands were visualized with SYBR Green I (Invitrogen, Carlsbad, CA), then excised and reamplified with the primer pair F338 (5′-ACTCCTACGGGAGGCAGCAG-3′) and R517. The resultant DNA fragments were purified with a MagExtractor kit (Toyobo, Osaka, Japan), and sequenced with the same primers using an ABI 3130 system and BigDye Terminator cycle sequencing kit, ver. 1.1 (Applied Biosystems, Foster City, CA). The sequence data were analyzed at the EZtaxon Server site [5] to seek the closest neighbors of the type strains.

### Detection of methanol emission from moss

In order to monitor methanol emission from the moss, we used a methylotrophic yeast strain, which expresses yellow fluorescent protein (YFP) in peroxisomes under the regulation of a methanol-inducible alcohol oxidase (AOX1) gene promoter (*Pichia pastoris* PPY12 (pYA005 and pYA006)) [6]. The yeast cells were cultivated in YNB (0.67% yeast nitrogen base without amino acids and 2% glucose) or YNM (0.67% yeast nitrogen base, 0.5% yeast extract and 1% methanol) at 28°C for 2 days. The YNB-grown cells were harvested and washed with water. A 5 µL aliquot of the suspension (OD600 = 1) was applied onto *R. japonicum* protonemata (pregrown for 1 week) [3] on Y medium. The suspension (5 µL) was also applied onto nonsterile, well-washed gametophytes of *R. japonicum* inoculated on Y medium. After incubation at 23°C for 1–5 days, the yeast cells were transferred onto glass slides and observed with a confocal laser scanning microscope (FV-1000, Olympus, Tokyo, Japan). Fluorescence of YFP (514 nm excitation and 527 nm emission) was observed. Yeast cells applied onto Y medium lacking plants and with or without 0.5% methanol were used as positive and negative controls, respectively.

### Isolation of methylotrophic bacteria

Since the DGGE analysis for the liquid culture samples revealed the predomination of methylotrophic bacteria, we tried to isolate methylotrophs. Samples from the liquid culture were spread on solidified methanol media [7]. The resultant pink-pigmented colonies were purified by restreaking them for three rounds on the same medium. Six pink-pigmented bacteria were isolated (Strains 11A and 11B (from sample A, 1 week), 21B and 21C (sample B, 1 week), 22A (sample B, 4 week) and 41A (sample D, 1 week)).

### Characterization of the isolates

For each of the six strains isolated, the 16S rRNA gene was sequenced, and the strains were characterized on the basis of their interaction with plants, as described previously [3]. *Methylobacterium extorquens* AM1 (ATCC 14718) was also tested. In brief, the strains were tested for nitrogen fixation on nitrogen-free medium [3] (glucose was substituted with 0.5% methanol), calcium phosphate solubilization [8], production of siderophore [9], cyanate [10] and indoleacetic acid [11], and utilization of sucrose, carboxymethyl cellulose, protein [12], chitin [13], pectin, starch [14] and β-glucan [12]. Where appropriate, glucose as carbon source was substituted with 0.5% methanol. Methanol dehydrogenase (MDH) activity was measured using cell-free extracts of cells grown on methanol medium [15]. Pyrroloquinoline-quinone (PQQ) content of the culture medium was quantified by HPLC as described previously [7]. Since methanol medium was not suited for PQQ production, we used PQQ medium, which has the following composition according to Urakami et al. [16]: 1 g (NH_4_)_2_SO_4_, 3 g KH_2_PO_4_, 0.15 g MgSO_4_·7H_2_O, 5 mg iron (III) citrate·nH_2_O, 8 mL methanol, 3 mg CaCl_2_·2H_2_O, 0.1 mg MnCl_2_·4H_2_O, ZnSO_4_·7H_2_O, CuSO_2_·5H_2_O, 0.01 mg KI, 0.01 mg (NH_4_)_6_Mo_7_O_24_·4H_2_O, 1 mg NaCl, and 1 mL of vitamin solution per liter that is used for methanol medium [7]. Enzymatic activity of 1-aminocyclopropane-1-carboxylate (ACC) deaminase was measured according to Penrose and Glick [17].

### Growth-promotion activity on mosses

The isolates were tested for plant-growth promoting activity toward aseptically grown *R. japonicum* protonemata [3]. The area of the elliptical protonemata colony was measured using Image J software [18]. The protonemata suspension was also inoculated onto Florialite (5-cm square and 2-cm high) that had been autoclaved and soaked in 1,000-fold diluted Hyponex (concentrated type). A washed bacterial suspension (OD = 1.0, 50 µl) was applied to the protonemata. The Florialite was placed on granulate reddish soil in Petri dishes and incubated at 22°C for 60 days under a fluorescent lamp with a continuous photon flux density of 100 µmol/m^2^/s^1^. As controls, one Petri dish without bacteria and another containing 50 µl of 3 mg/L kinetin, were prepared.

Strain 22A was also tested on other bryophytes. Sterile protonemata of *Haplocladium microphyllum*, *Trachycystis microphylla*, and *Bryum* sp. were inoculated on Y medium and a colony of strain 22A that had formed on methanol medium was inoculated onto the protonemata. The protonemata were allowed to grow under the same conditions as *R. japonicum*.

### Growth promotion of seed plants

Seeds of *Nicotiana tabacum*, *Nicotiana benthamiana*, *Arabidopsis thaliana* (ecotype Columbia), *Glycine max* cv. Enrei, and *Oryza sativa* ssp. *japonica* cv. Nipponbare were sterilized with 70% ethanol for 1 min, then treated with 1–2% sodium hypochlorite solution containing 0.3% Tween 20 for 5–20 min. The seeds were washed with sterile water 5 times.

Ten *Nicotiana* seeds were placed on filter paper in a Plant box™ (Iwaki, Tokyo, Japan, 5.5-cm square and 9-cm height) and soaked with 5-mL liquid 1/2 Murashige-Skoog (MS) medium (Sigma). Bacterial cells (500 µL, OD = 1.0) grown in methanol medium and washed with sterile water were added to the solution. The seeds were allowed to grow at 25°C (12 h light/12 h dark) for 15 days. Ten *A. thaliana* seeds were placed on solidified 1/2 MS medium (0.8% agar) prepared in a Petri dish (9-cm diameter); each seed received 5 µL of bacterial suspension, and was grown at 23°C (12 h light/12 h dark) for 10 days. Seven *G. max* seeds were placed on 40-mL 0.8% agar prepared in a Plant box™; each seed received 10 µL of the suspension, and was grown at 25°C for 7 days (16 h light/8 h dark). Fifteen *O. sativa* seeds were grown as *G. max* except for 8 days (12 h light/12 h dark). These preparations were made in triplicate. When the plant seedlings reached their maximal sizes allowed by the containers, which typically occurred during growth periods specified above, the length of root and shoot and the fresh weight of the seedlings were measured.

### Practical cultivation of *R. japonicum*

#### Liquid culture system

Adult gametophytes of *R. japonicum* (n = 100, length 1±0.3 cm) were put in 900-mL tap water. A culture of strain 22A (grown in methanol medium for 7 days, OD600 = 0.71, 25 mL) was added to the *R. japonicum* culture. A control with tap water (25 mL) added instead of the culture and another with uninoculated methanol medium (25 mL) were prepared. The experiment was done in triplicate. The plants were allowed to grow at 25°C at a photon flux density of 100 µmol/m^2^/s under a fluorescent lamp with a 16 h light/8 h dark cycle. Air was bubbled in the flask at 3 L/min. After 1, 2, and 3 weeks of cultivation, the number of branches per plant were counted, and apical growth was measured.

#### Field experiment

Adult gametophytes of *R. japonicum* (30 g, equivalent to 26 g dry weight, with a length of 5.6±1.4 cm), which had been grown in liquid culture for 4 weeks and then incubated in a greenhouse for 4 weeks, were placed on plastic trays (32×42×3 cm). The trays were prefilled with different support materials, i.e. lime soil, Yamakusari soil (a heavily weathered mountain rock soil, on which native *R. japonicum* preferentially grows in Shiga Prefecture, Japan), Kanuma soil (weathered pumice stone), vermiculite, rock wool, and plastic net. One of the two trays prepared for each support was sprayed with a suspension of strain 22A grown in methanol medium (0.8×10^8^ CFU/mL, 100 mL). The trays were placed in an experimental field belonging to Meiho Construction (Shiga, Japan) for 12 weeks (Oct. 22, 2010 to Jan. 13, 2011). The plants were allowed to grow without any treatment. The length of 50 randomly selected plants was measured before and after growth in the field.

### Nucleotide accession numbers

The GenBank/EMBL/DDBJ accession numbers for the 16S rRNA gene sequences reported in this paper are DM377424 (strain 11A), DM377425 (strain 11B), DM377428 (strain 21B), DM377429 (strain 22A), and AB558142 (strain 41A).

## Results and Discussion

### Predomination of methylotrophic bacteria in liquid culture

In a previous study, we isolated bacteria that belonged mainly to *Duganella*, *Pseudomonas*, and *Rhodococcus* species from liquid cultures of *R. japonicum* [3]. Some of these bacteria promoted the growth of moss protonemata, possibly through plant hormone synthesis. Here we used culture-independent techniques to reveal the microbial community structures of the same liquid cultures to obtain more insight into the interaction between *R. japonicum* and the bacteria detected in the cultures.

**Figure 1:**
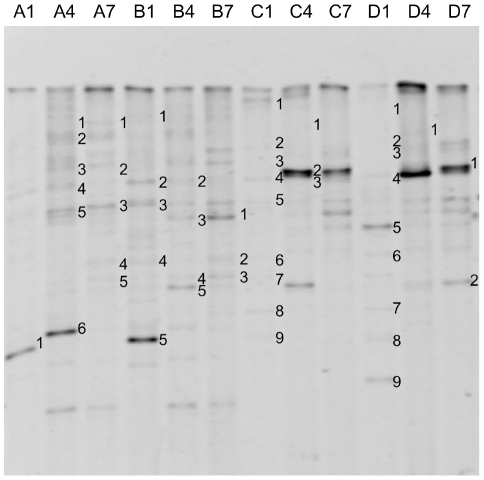
DGGE profiles showing the bacterial communities of *R. japonicum* liquid cultures. A–D indicate the growth conditions (see text) and 1, 4 and 7 refer to the week of sampling. Bands indicated with numbers were purified and sequenced (for results, see Table 1).

We examined the microbial community structure in samples of *R. japonicum* liquid cultures by means of DGGE (Fig. 1 and Table 1). The DNA sequences of selected fragments labeled in Fig. 1 were determined after gel-excision and re-amplification by PCR. Several fragments indicated in Fig. 1 yielded mixed sequence data, suggesting that they originated from more than one bacterial species. We therefore excluded them from our analysis and these fragments are not listed in Table 1. We found the predomination of methylotrophs such as *Methylobacterium* species (*M. tardum*, and *M. zatmanii)*, *Methylovirgula ligni*, *Methylocella tundrae*, and *Methylocapsa aurea*. Besides them, *Sphingomonas* species [19], *Pseudomonas* and *Achromobacter* species [20], *Bacillus* and *Micrococcus* species [21] were also detected. These genera were recently shown to contain methylotrophic strains, although it is unknown whether the detected bacteria are really methylotrophic or not only by DGGE analysis. These results suggested that methanol (or other C1-compounds) could be a main carbon source in liquid cultures. Because we used nutrient-poor media for cultivation of the moss, the carbon source for heterotrophic bacteria must have been supplied from the plant. Plants can emit various C1-compounds including methanol [22], [23], chloromethane [24], and methane [25], [26]. We confirmed methanol emission from the moss as described below.

**Table 1:** Result of DGGE analysis for *R. japonicum* liquid culture samples.

DGGE band	Closest type strain	Strain	GenBank accession no.	Pairwise similarity (%)
A1-1	*Micrococcus terreus*	V3M1(T)	FJ423763	98.3
A4-1	*Mycoplasma ravipulmonis*	Mus musculus	AF001173	89.1
A4-4	*Sphingopyxis witflariensis*	W-50(T)	AJ416410	92.1
A4-5	*Rhizomicrobium electricum*	Mfc52(T)	AB365487	94.8
A4-6	*Spirosoma linguale*	DSM 74(T)	CP001769	97.5
A7-4	*Methylobacterium tardum*	RB677(T)	AB252208	96.5
B1-3	*Micrococcus terreus*	V3M1(T)	FJ423763	99.2
B1-5	*Micrococcus terreus*	V3M1(T)	FJ423763	100
B4-3	*Methylovirgula ligni*	BW863(T)	FM252034	96.6
B4-4	*Methylobacterium zatmanii*	DSM 5688(T)	AB175647	96.9
B4-5	*Achromobacter marplatensis*	B2(T)	EU150134	96.4
B4-6	*Sneathiella glossodoripedis*	IAM 15419(T)	AB289439	82.9
C1-1	*Spirosoma linguale*	DSM 74(T)	CP001769	94.1
C1-5	*Acinetobacter haemolyticus*	DSM 6962(T)	X81662	93.7
C1-6	*Achromobacter marplatensis*	B2(T)	EU150134	93.2
C1-7	*Methylocella tundrae*	T4(T)	AJ555244	92.1
C1-8	*Sanguibacter keddieii*	DSM 10542(T)	CP001819	99.2
C1-9	*Streptomyces thermovulgaris*	NBRC 16609(T)	AB249975	99.2
C4-1	*Cytophaga hutchinsonii*	ATCC 33406(T)	CP000383	100
C4-2	*Pedobacter koreensis*	WPCB189(T)	DQ092871	84.2
C4-5	*Giesbergeria sinuosa*	LMG 4393(T)	AF078754	84.4
C4-6	*Reyranella massiliensis*	URTM1(T)	EF394922	100
C7-1	*Hirschia maritima*	GSW-2(T)	FM202386	96.6
C7-2	*Methylocella tundrae*	T4(T)	AJ555244	96.5
C7-3	*Verminephrobacter eiseniae*	EF01-2(T)	CP000542	85.2
D1-1	*Sphingopyxis witflariensis*	W-50(T)	AJ416410	98.3
D1-2	*Bacillus pocheonensis*	Gsoil 420(T)	AB245377	92.9
D1-3	*Methylocapsa aurea*	KYG(T)	FN433469	98.6
D1-4	*Methylocella tundrae*	T4(T)	AJ555244	97.4
D1-5	*Salinibacterium xinjiangense*	0543(T)	DQ515964	100
D1-6	*Dyadobacter psychrophilus*	BZ26(T)	GQ131577	96.9
D1-7	*Zimmermannella bifida*	IAM 14848(T)	AB012595	80.5
D1-9	*Dyadobacter psychrophilus*	BZ26(T)	GQ131577	97.5
D4-1	*Spirosoma linguale*	DSM 74(T)	CP001769	95.7
D7-1	*Polysiphonia harveyi* Chloroplast		AY731510	87
D7-2	*Polaromonas aquatica*	CCUG 39402(T)	AM039830	97

Identification was done at EZtaxon site (version 2.1). The closest relatives are listed.

### Methanol emission from *R. japonicum*

**Figure 2:**
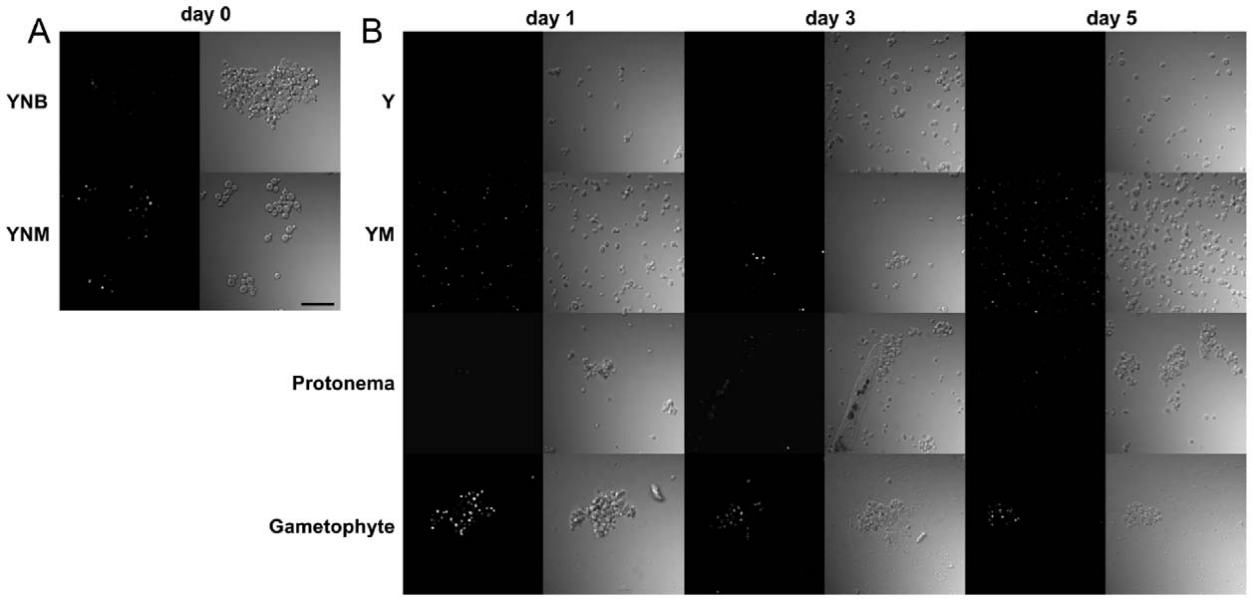
Detection of methanol emitted from *R. japonicum.* A. Control observation with *P. pastoris* PPY12 (pYA005 and pYA006) grown on YNB and YNM for 2 days. B. YNB-grown cells were applied onto Y medium, Y+0.5% methanol (YM); protonemata were grown on Y medium and non-sterile gametophytes were inoculated on Y medium. Left panel, fluorescence; right panel, differential interference contrast images. Bar, 20 µm.

Using *P. pastoris* PPY12 (pYA005 and pYA006), we detected methanol emission from gametophytes and protonemata of the moss. As shown in the control experiment (Fig. 2A), only methanol (YNM)-grown cells emitted fluorescence in their peroxisomes. Yeast cells applied on Y medium without plants did not emit fluorescence, and those on Y+0.5% methanol emitted fluorescence within 1–5 days. Yeast cells applied onto protonemata exhibited fluorescence for 5 days, with a weaker intensity than on Y+0.5% methanol, and yeast cells applied to gametophytes exhibited stronger YFP signals (Fig. 2B) than those on protonemata. These results suggested that methanol is emitted from both gametophytes and protonemata of *R. japonicum*. Although methanol is considered to be emitted from plant stomata, since methanol is water-soluble and the cuticular surface of plants is hydrophobic [23], [27], detection of methanol was recently reported on epidermal cells of *Arabidopsis* leaves [28]. In many cases, the leaves of bryophytes consist of a single cell layer that is covered with cuticle and these leaves do not contain any stomata. Thus methanol is suspected to be leaking from the moss plant surface, as water can be absorbed through the entire body of the plants.

### Characterization of *Methylobacterium* species

Most of the colonies that formed on methanol medium using the liquid culture samples were pink, suggesting that they belong to *Methylobacterium* species. Six strains were isolated and identified as *Methylobacterium* species based on their 16S rRNA genes sequences. The strains 11A, 11B, 21B, and 21C had *M. extorquens* (NCIMB9399^T^, AB175633) as the closest phylogenetic relative, strain 22A was closest to *M. aquaticum* (CCM7218^T^, AJ635303) and strain 41A was closest to *M. oryzae* (DSM18207^T^, AY683045) (Fig. S1). Their biochemical characteristics are summarized in Table S1, using *M. extorquens* strain AM1 for comparison. Some of the strains were able to produce indoleacetic acid [29], [30], siderophore [31], ACC deaminase [32] and hydrogen cyanate [10]. Solubilization of calcium phosphate was also confirmed [33], [34]. PQQ is a key compound produced by rhizobacteria that promotes plant growth [35]. Interestingly, PQQ is the cofactor of MDH in *Methylobacterium* species, and MDH expression in the phyllosphere has been confirmed by proteomic analysis [36]. The PQQ production level was improved by using PQQ medium [16]. All *Methylobacterium* species isolates had ACC deaminase activity, at a level that seemed high for plant-growth promoting rhizobacteria [17]. These characteristics are considered important for beneficial interaction between microbes and plants, although it is not clear which of the traits is crucial for the growth promotion effect described below.

### Growth promotion of mosses

**Figure 3:**
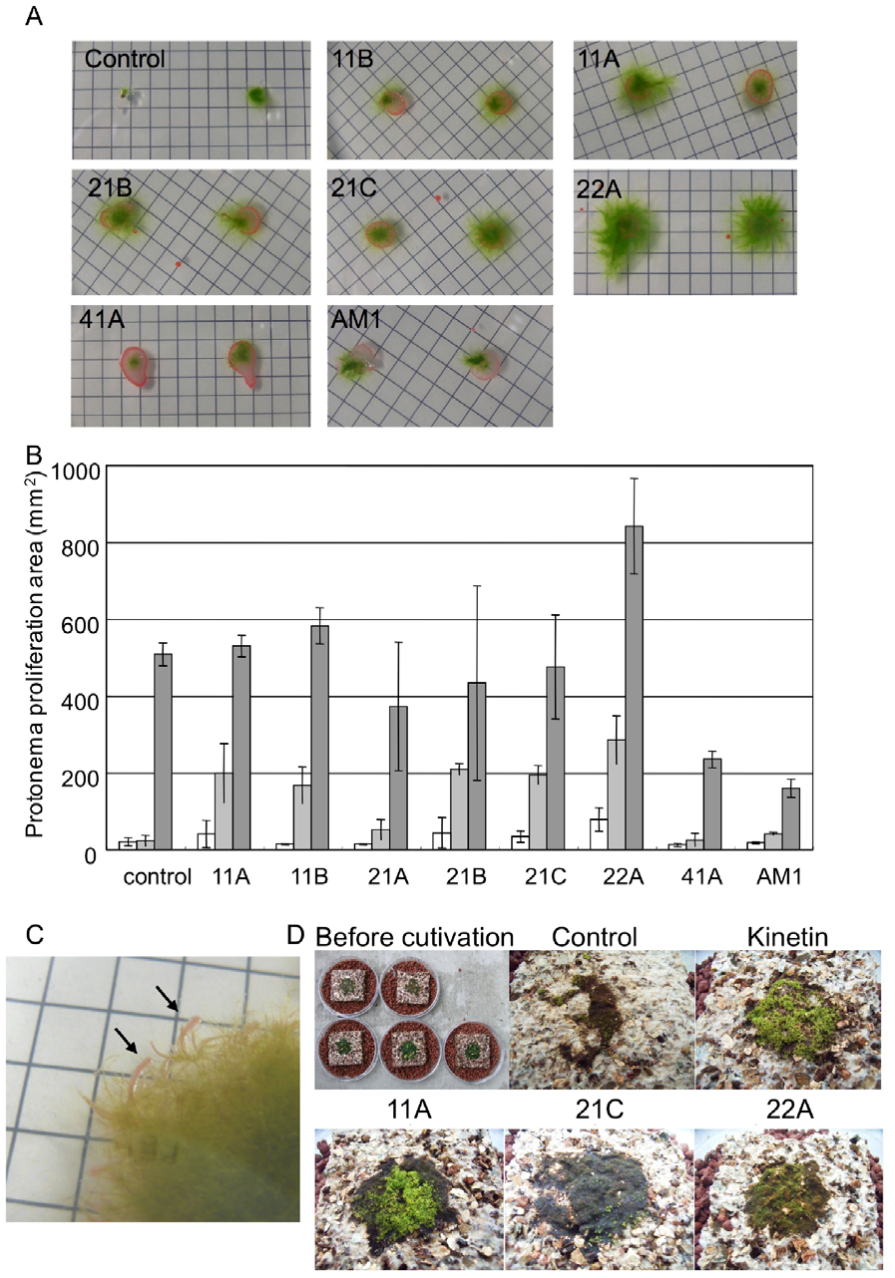
Growth promotion of *R. japonicum* by *Methylobacterium* isolates. A, Protonemata growth on Y medium in the presence of bacterial isolates (35 days of cultivation). Grid, 5 mm. B, Quantification of protonemata growth. White bars, 22 days; light gray bars, 35 days; and gray bars, 63 days. Growth was evaluated by measuring the area of protonemata proliferation, regarded as ellipses. Error bars, standard deviation (n = 8). C, Strain 22A growing with protonemata on Y medium. Grid, 5 mm. D, Induction of gametophyte formation by application of bacterial isolates. A suspension of *R. japonicum* protonemata was inoculated on Florialite, to which 50 µL of water (control), kinetin (3 mg/L), or a bacterial suspension (11A, 21C and 22A) was applied. Images were taken after 60 days of cultivation.

The isolated *Methylobacterium* species were applied onto sterile protonemata of *R. japonicum*, and an obvious growth-promotion effect was observed, as shown in Fig. 3A. Among the isolates, strain 22A showed the greatest effect as quantified in Fig. 3B. In addition, as the protonemata grew on solid Y medium, growth of the applied bacterial strains was clearly visible around the protonemata (Fig. 3C). These isolates could not grow in isolation on the medium since Y medium contains no carbon source, and methanol supplementation supported their growth (data not shown). Thus it is likely that the plant supplied a carbon source, possibly including methanol, to the isolates. The protonemata of *R. japonicum* rarely develop into gametophytes on agar plate media. Formation of gametophytes was observed when the protonemata were grown on Florialite. The gametophyte development was promoted by inoculation with the isolates, an effect equivalent to that in an experiment with kinetin added instead (Fig. 3D). These results suggest that *Methylobacterium* species not only promoted the growth but also the development of gametophytes, possibly through synthesis of phytohormones, including auxin and cytokinin. It has already been reported that growth and bud formation of a moss (*Funaria hygrometrica*) [37] and growth of liverworts (*Marchantia polymorpha* L. and *Lunularia cruciata* L.) [38] are promoted by *Methylobacterium* species, and the importance of their phytohormone synthesis is suggested [37], [39], [40]. In this study, we detected auxin production (Table S1) but did not investigate cytokinin production. We are currently trying to quantify the phytohormones produced by *Methylobacterium* species.

**Figure 4:**
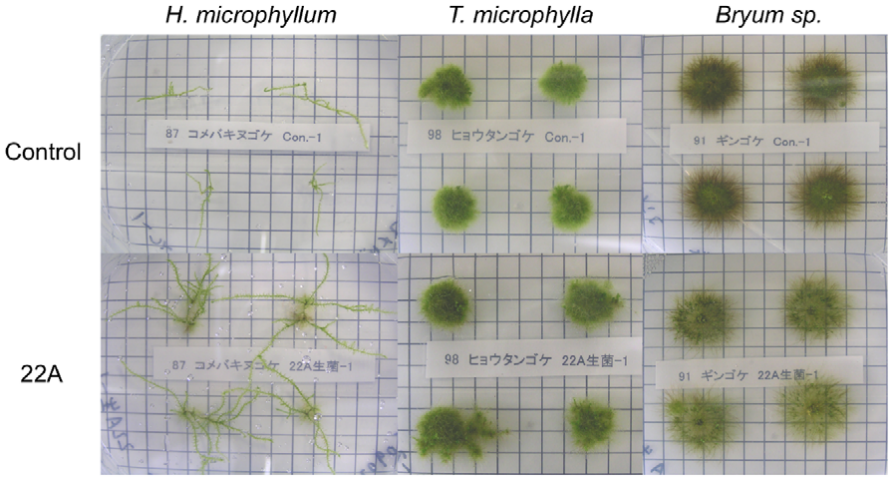
Effect of strain 22A application on other bryophytes. Strain 22A was applied onto protonemata of *H. microphyllum*, *T. microphylla*, and *Bryum* sp., and the plants were grown for 38 days on Y medium. The plants that received no bacterial cells served as controls. Grid, 5 mm.

Strain 22A was also applied onto protonemata of other bryophytes. Among those tested, the growth promotion of *H. microphyllum* was the most pronounced, as shown in Fig. 4. The growth of *T. microphylla* was stimulated only slightly by strain 22A. The density of the *Bryum* sp. colony was lowered by bacterial application. Thus, although growth conditions have not been optimized for the different bryophytes tested in this study, these results may indicate that growth promotion by *Methylobacterium* species is dependent on the moss-bacterium combination. Cultivation site and plant species are important determinants of *Methylobacterium* community composition in the plant phyllosphere [41], and plant species and soil type shape the structure and function of microbial communities in the rhizosphere [42]. Recently it was shown that an a-proteobacteria subpopulation in soybean is drastically affected by different nodulation phenotypes and amount of nitrogen fertilization [43]. These observations implicate the environment, plant species, and plant nutrient conditions as influencing the microbial community structure on plant surfaces. Thus, in order to promote plant growth, it will be important to use the microorganisms that can best grow under given conditions.

### Growth promotion of seed plants

We also tested the ability of the selected isolates to promote the growth of seed plants (Table 2). Strain 22A was effective on *A. thaliana*. The growth of *N. tabacum* was promoted by all tested strains. *N. benthamiana* growth was promoted only by strain 22A, and the application of strains 21B and 21C resulted in decreased seedling weight. Strain 22A promoted *O. sativa* growth. The seedling weight of *G. max* was increased by application of strains 21B and 21C. No statistically significant growth promotion was observed for *H. vulgare* with these three strains (data not shown). These results suggested that *Methylobacterium* species isolated from the moss culture were also effective in promoting growth of the tested seed plants, although there was again some combination specificity. It has been reported that *M. mesophilicum* can promote the growth of liverworts but not sunflower or maize [38]. On the other hand, isolates of *M. extorquens* were effective in promoting growth of various seed plants [27]. It is unknown whether this non-uniform effectiveness is due to plant-microbe specificity or the different experimental conditions of each study. The first would suggest that for practical application and promotion of growth, more plant species and bacterial strains will have to be examined to develop *Methylobacterium* as a general growth promoting strategy in plants. Field level experiments will also be important for checking the effect and practical application of our isolates in natural conditions.

**Table 2:** Growth promotion of seed plants by added *Methylobacterium* species.

			Strain name of isolated *Methylobacterium* species		
		Control (water)	21B	21C	22A
*Arabidopsis thaliana* (n = 30)	Root length (cm)	1.1±1.3	2.2±1.6*	1.8±1.6	2.1±1.8*
	Fresh mass (mg)	18±9.0	23±13.5	22±11.5	29±13.6**
*Nicotiana tabacum* (n = 30)	Shoot length (cm)	0.52±0.10	0.55±0.10	0.53±0.05	0.78±0.16**
	Root length (cm)	1.0±0.42	0.90±0.24	1.3±0.28**	1.1±0.34
	Fresh mass (mg)	41±20.3	58±17.6**	75±11.1**	70±19.5**
*Nicotiana benthamiana* (n = 30)	Shoot length (cm)	0.51±0.36	0.42±0.11	0.53±0.10	0.53±0.13
	Root length (cm)	1.2±0.78	1.1±0.45	1.4±0.32	1.1±0.30
	Fresh mass (mg)	44±28.0	22±8.2**	26±8.4**	66±12.0**
*Oryza sativa* (n = 45)	Shoot length (cm)	4.1±1.01	nt	3.7±0.98	3.9±0.75
	Root length (cm)	10±3.1	nt	8.8±3.2	9.4±3.4
	Fresh mass (mg)	61±12.4	nt	64±12.6	67±11.5*
*Glycine max* (n = 21)	Shoot length (cm)	7.7±1.9	8.3±0.9	8.1±1.6	8.1±2.1
	Root length (cm)	9.8±3.3	11±1.4	12±3.4	7.1±3.1*
	Lateral root number	26±9.9	37±6.8**	39±12.3**	28±10.3
	Fresh mass (g)	1.4±1.25	1.5±0.18*	1.6±0.31*	1.4±0.24

Data presented as mean ± SD. Fresh mass indicates that of entire plants.

nt: not tested.

* , p<0.05 and.

** , p<0.01 (Student's T-test).

### Practical cultivation of *R. japonicum*

**Figure 5:**
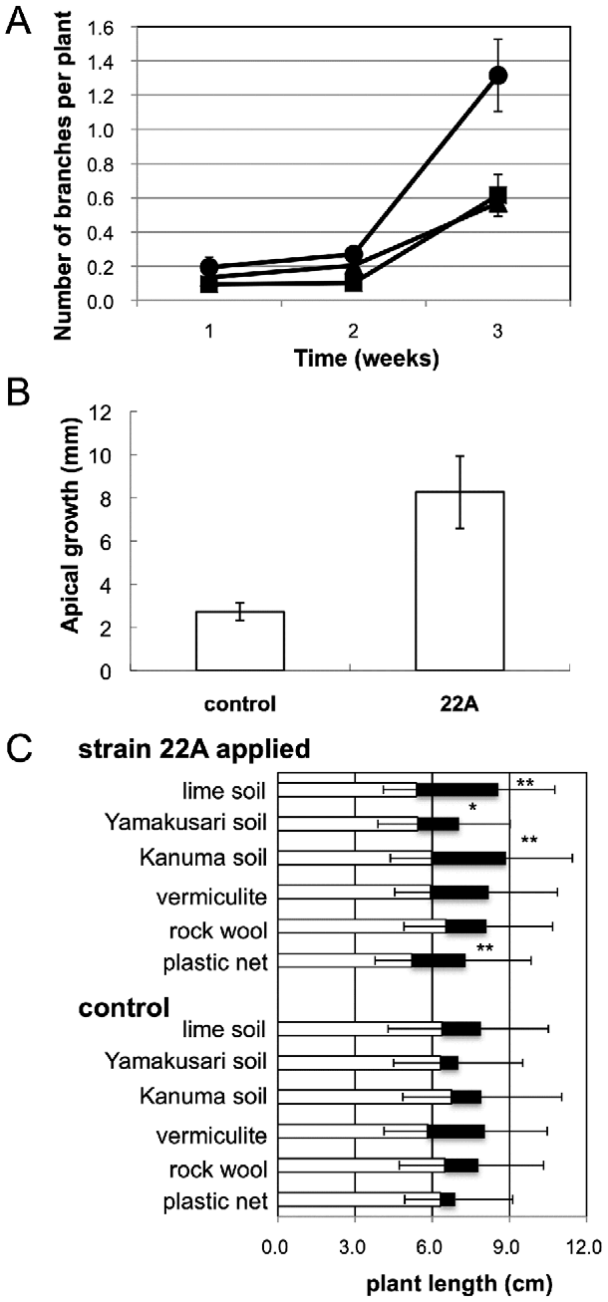
Application of strain 22A to *R. japonicum*. A and B, growth-promotion experiment of *R. japonicum* in hydroponic culture. A, branch number per plant during cultivation. Circle, 22A-applied sample; triangle, methanol medium; and square, control (water). Data are presented as mean ± SEM (four replicates of n = 100). B, apical growth at the end of cultivation. Data are presented as mean ± SEM (four replicates of n = 100). C, gametophyte growth of *R. japonicum* in a field experiment using different support materials. White bar, average size of the plants before the experiment (bar, standard deviation only to left side); and black bar, increase in size after the experiment (bar, standard deviation only to right side). Statistical significance of the difference between control and 22A-treated samples is shown as * (p<0.05) and **(p<0.01) (Student's t-test, n = 50).

In our process for *R. japonicum* production, branching is first stimulated during cultivation in liquid, and the branched gametophytes are then placed on trays and cultured in a greenhouse. Since the stimulation of branching is a crucial step in the liquid cultivation step, we evaluated the effect of strain 22A application on branch formation. Apical growth also occurs in this step, which is stimulated more in the greenhouse. As shown in Fig. 5(A), the application of strain 22A clearly stimulated branching in 3 weeks compared to control experiments with either water or methanol medium (p<0.00001). Apical growth was also clearly stimulated by strain 22A application compared to the non-inoculated control (Fig. 5(B)) (p<0.00001). Addition of methanol medium was not effective, although we expected indirect effects due to the predomination of methylotrophs. These results clearly indicated that the application of strain 22A cells in liquid culture stimulates branching and apical growth of the moss.

After stimulation of branching in liquid culture (4 weeks), the plants were allowed to grow in a greenhouse to adapt to the environment (4 weeks). The plants then had to be fixed onto support materials. There are several methods to fix the plants on trays that are later settled on roofs. In this experiment we used vermiculite, Kanuma soil, Yamakusari soil, and lime soil, all of which have good drainage and may have a nutritional effect. We also used plastic net and rock wool, which can fix the plants but have no nutritional effect. Fig. 5C shows growth stimulation of gametophytes of *R. japonicum* by application of strain 22A in a field experiment. Since it was difficult to make the plant size uniform prior to the test, the average size before the experiment showed some variation (white bar). In the control experiment (in the absence of strain 22A), vermiculite and lime soil showed good performance in increasing plant size (23–38% increase). Although it is claimed that mosses require a little nutrient, support on plastic net and rock wool, with no nutrients, led to poor growth. In the strain 22A-treated samples, an increase in plant length was clear. Among the soils tested except for vermiculite, the application of strain 22A significantly increased the growth of the moss compared to the control. Kanuma soil is considered to be a good support material, because of its light weight, low cost, porousness, stability and ease of handling. By using Kanuma soil, strain 22A doubled the moss growth rate, which resulted in reducing the cultivation time by half. This is quite an advantage in cutting the cost and time to produce a moss carpet. We observed, however, moss grown this way is not resistant to strong wind at roof level, and other plant species disseminated by wind-borne seeds tend to grow on the soil. Support materials that can overcome these problems are still needed.

Here we report a unique methylotroph-predominating community structure in liquid cultures of *R. japonicum*. The moss emits methanol, which is considered to be the main carbon source supplied to the microbes in the liquid culture. We also showed strong plant-growth promotion activity of the isolated *Methylobacterium* species, and the growth-promotion effect was specific to the plant-microbe combination. It is not yet known which biochemical ability of the isolates is crucial for growth promotion. The methanol emission from the moss and growth promotion by *Methylobacterium* species are considered to result from their mutualistic symbiosis [40]. Our isolates, especially strain 22A, have great potential for application to industrial production of the moss as a roof greening material and as biofertilizers for crops. Feasibility studies are now in progress in our laboratory.

## Supporting Information

Figure S1 **Phylogenetic analysis based on 16S rRNA gene sequences constructed after multiple alignment of data (1290 nt) and clustering with neighbor-joining method.** Bootstrap values greater than 70% based on 1000 replications are listed as percentages at the branching points. The scale bar indicates the number of substitutions per nucleotide position. The sequence of *Rhodopseudomonas palustris* DSM 123^T^ was used as an outgroup.
(PPT)

Table S1 **Characterization of** ***Methylobacterium*** **isolates.**
(XLS)
