# Supplementary figures and images for: Practical Application of Methanol-Mediated Mutualistic Symbiosis between Methylobacterium Species and a Roof Greening Moss, Racomitrium japonicum
Source: PLoS One. 2012 Mar 29;7(3):e33800. doi: 10.1371/journal.pone.0033800 (PMC3315585; doi:10.1371/journal.pone.0033800)

## Slide 1
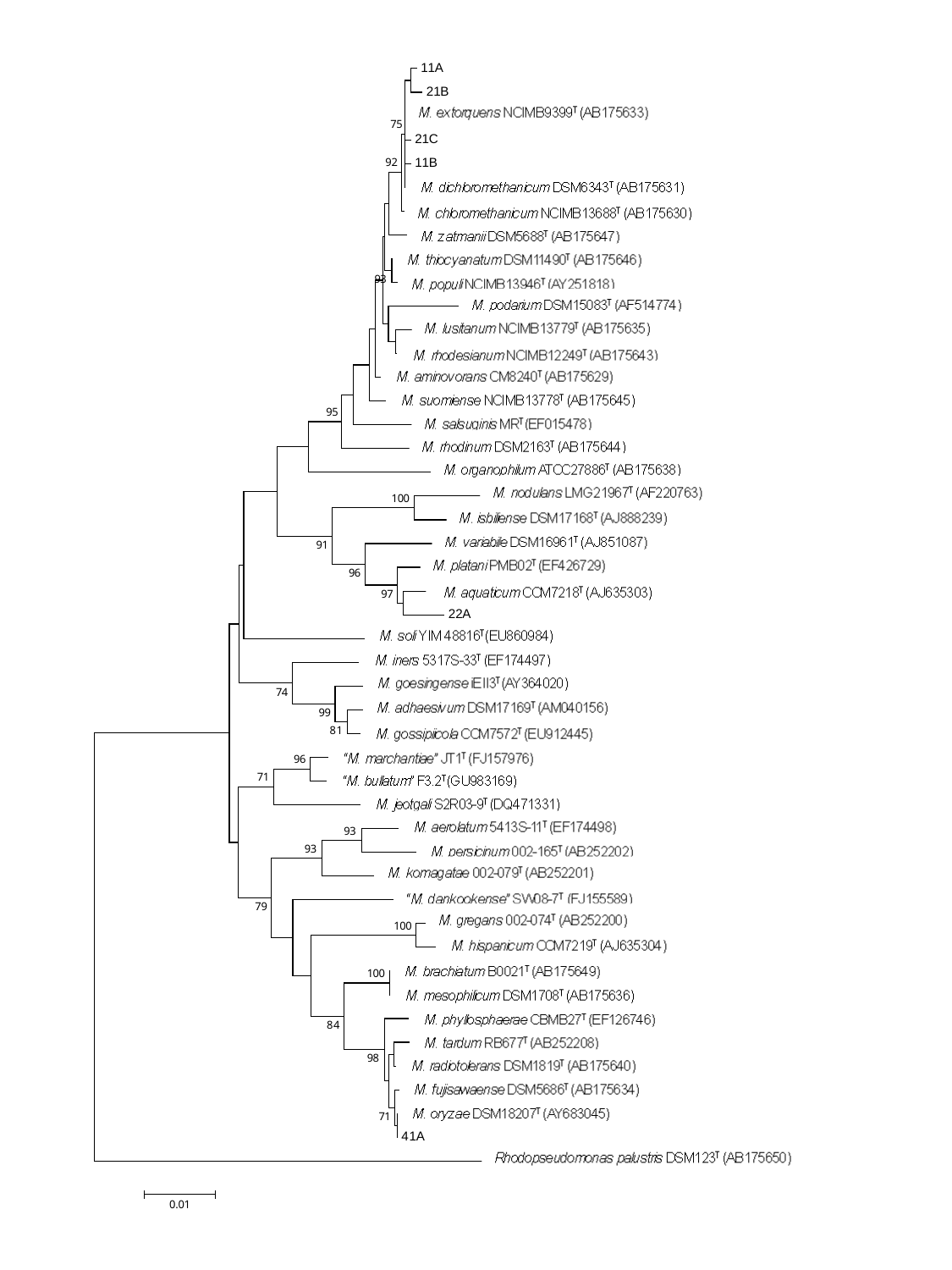

Supplement: Figure S1 — Phylogenetic analysis based on 16S rRNA gene sequences constructed after multiple alignment of data (1290 nt) and clustering with neighbor-joining method. Bootstrap values greater than 70% based on 1000 replications are listed as percentages at the branching points. The scale bar indicates the number of substitutions per nucleotide position. The sequence of Rhodopseudomonas palustris DSM 123T was used as an outgroup. (PPT) [file pone.0033800.s001.ppt]
